# Two successful decades of Swiss collaborations to develop new anti-malarials

**DOI:** 10.1186/s12936-019-2728-8

**Published:** 2019-03-22

**Authors:** Rob Hooft van Huijsduijnen, Timothy Wells, Marcel Tanner, Sergio Wittlin

**Affiliations:** 1 Medicines for Malaria Venture, Route de Pré Bois 20, 1215 Geneva, Switzerland; 2 Swiss Tropical & Public Health Institute, Socinstrasse 57, 4002 Basel, Switzerland; 3 University of Basel, Basel, Switzerland

**Keywords:** Malaria, Drug discovery, Product-development-partnership, Switzerland

## Abstract

Over the last two decades there has been a renaissance in the pipeline of new drugs targeting malaria, with the launch of new products that help save the lives of children throughout the world. In addition, there is a wealth of new molecules both entering and progressing through clinical development. These bring hope for a new generation of simpler and more effective cures that could overcome the emerging threat of drug resistance. In addition, there is hope that some of these medicines will have prophylactic activity and can be used to protect vulnerable populations, given the absence of a highly effective vaccine. Switzerland has played a key role in the development of these medicines. First, the country has a long history of understanding the biology of parasites and the pharmacology of drug responses through the leadership of the Swiss Tropical and Public Health Institute in Basel. Second, the highly successful Swiss pharmaceutical industry brings, beyond excellence, a strong interest in neglected diseases, building on work at Hoffmann-La Roche in the last century and with more recent products from Novartis and other Swiss companies. Third, the emergence of product-development-partnerships, in this case led by the Medicines for Malaria Venture, based in Geneva, has helped to catalyze the development of new medicines and bring the community together within Switzerland and beyond. Finally, this progress would not have been possible without the engagement of the Swiss people and the support of the federal government through the Swiss Agency for Development and Cooperation (SDC), the State Secretariat of Education, Research and Innovation (SERI) and the Swiss Republic and Canton of Geneva.

## Background

**Fig. 1:**
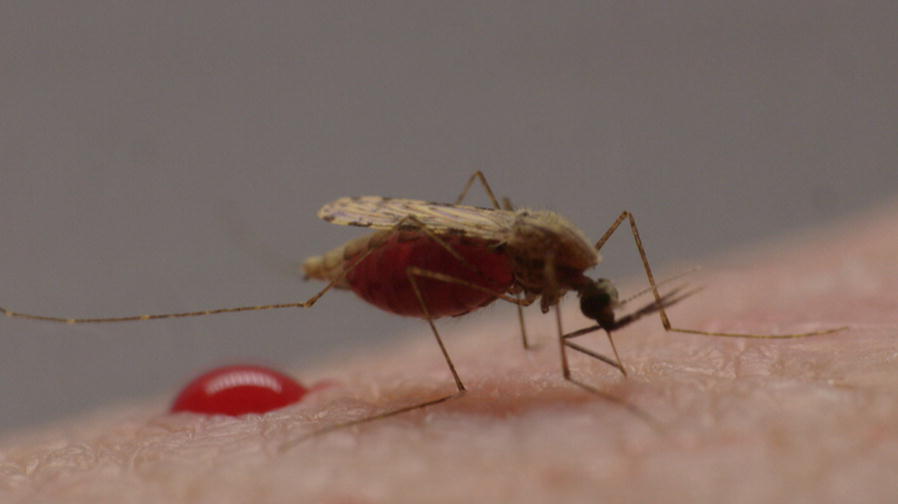
An *Anopheles* mosquito taking a human blood meal. Photo credit: Mary Soan, SMG Photo Contest

**Fig. 2:**
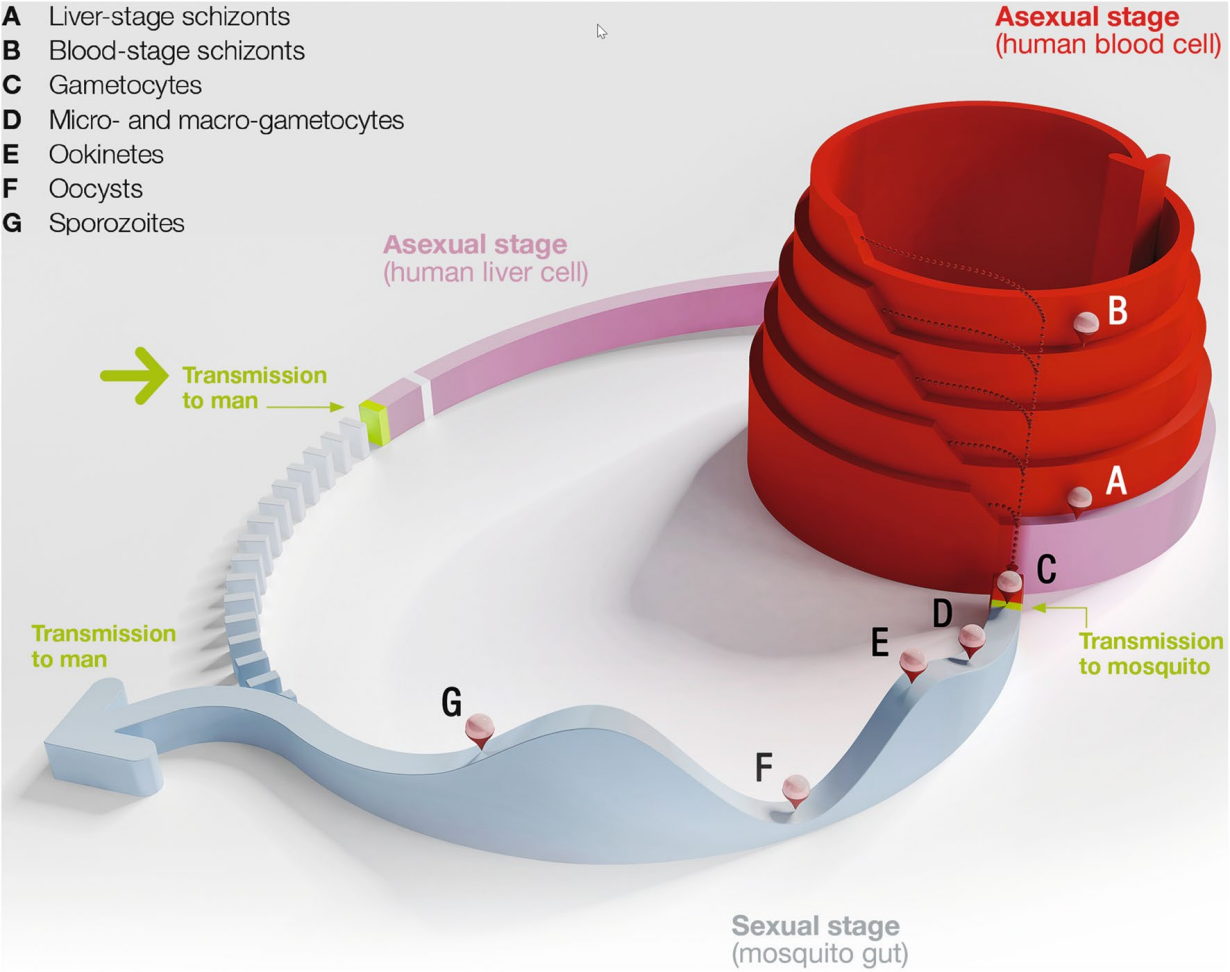
Schematic overview of stages (**A**–**G** top left) in the malaria parasite’s (*Plasmodium*) lifecycle. The Z-axis (above the plane) measures parasite numbers

Malaria is caused by protozoan parasites that are transmitted by mosquitoes (See Figs. 1 and 2 for the parasite’s life cycle, and [1]). Malaria remains one of the deadliest infectious diseases, with an estimated 435,000 deaths in 2017, in spite of a decrease by 20% between 2010 and 2017 [2, 3]. Switzerland’s successes in the fight against malaria are based on a variety of circumstances, some of which have deep historical roots.

First, as part of the country’s long-standing commitment to international cooperation, Geneva has hosted the World Health Organization (WHO) since its inception in 1946. Along with other organizations within the United Nations, the WHO monitors global health needs and sets priorities and treatment guidelines, including for malaria. The WHO maintains close links with the International Red Cross that was founded by Henry Dunant in the same city, in 1863.

**Fig. 3:**
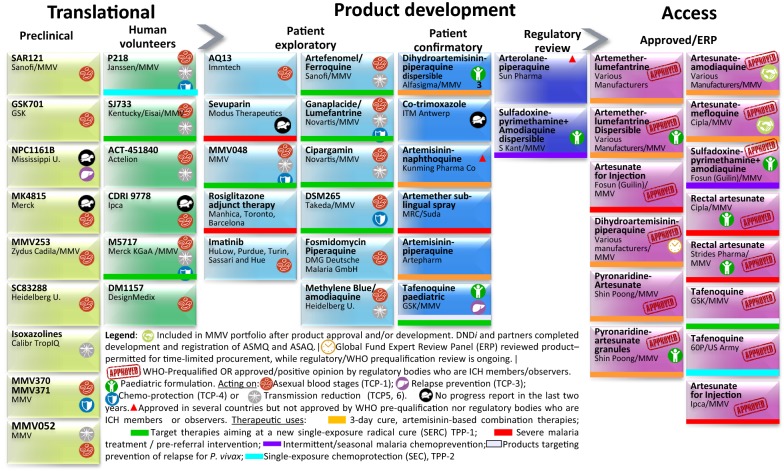
The antimalarial pipeline with key players in R&D for malaria drug discovery in Switzerland

Second, the country has a longstanding excellence in science. Among countries with over 1 M inhabitants, Switzerland has the highest per-capita ranking of science Nobel Prizes. High-quality malaria research is being carried out at universities in Basel, notably the Swiss Tropical and Public Health Institute (Swiss TPH), Geneva, Lausanne, Berne, Lugano and Zurich (Fig. 3), with over 270 ‘Swiss’ scientific publications in 2017 (as listed by Scopus, Elsevier B.V., based on the corresponding authors’ addresses). Work at Universities is aimed at a better understanding of malaria, including new targets and small molecules, but also the immunology of the disease. Important work in the last area is done by the team of Prof. Lanzavecchia at the University of Lugano [4, 5]. Such work helps in the development of better vaccines, but may also lead to therapeutic antibodies [6].

Third, Switzerland is a leader of pharmaceutical innovation; in 2017 it exported $41 billion worth of medicines, ranking second among countries (of any size; [7]). As further exemplified below, several of these highly successful companies, notably Novartis, are actively engaged in malaria, a disease generally considered of humanitarian rather than investor interest.

Fourth, in order to join forces between commercial drug discovery entities and not-for-profit organizations the innovative public private partnership (PPP) model for malaria was pioneered in Switzerland by Medicines for Malaria Venture (MMV) and later pursued by Drugs for Neglected Diseases *Initiative* (DND*i*)—organizations both based in Geneva. Such partnerships are essential to advance new potential medicines through clinical development, aligned with the global needs (the target compound profiles [6, 8, 9] and the eradication agenda [10]) for malaria.

Fifth, Switzerland has traditionally been open to attracting talent. It ranks significantly higher than its neighbours (Germany, France and Italy; http://tinyurl.com/zaubzgw) in its proportion of foreign-born individuals. Switzerland is playing an active and strategic role on the global level to promote health as a global public good and a universal human right. The Swiss Agency for Development and Cooperation (SDC) has been a strong supporter to MMV’s mission since its inception in 1999, for which MMV is extremely grateful. MMV has been specifically focused on discovering, developing and delivering new medicines for malaria.

## Switzerland’s role in anti-malarials available today

**Fig. 4:**
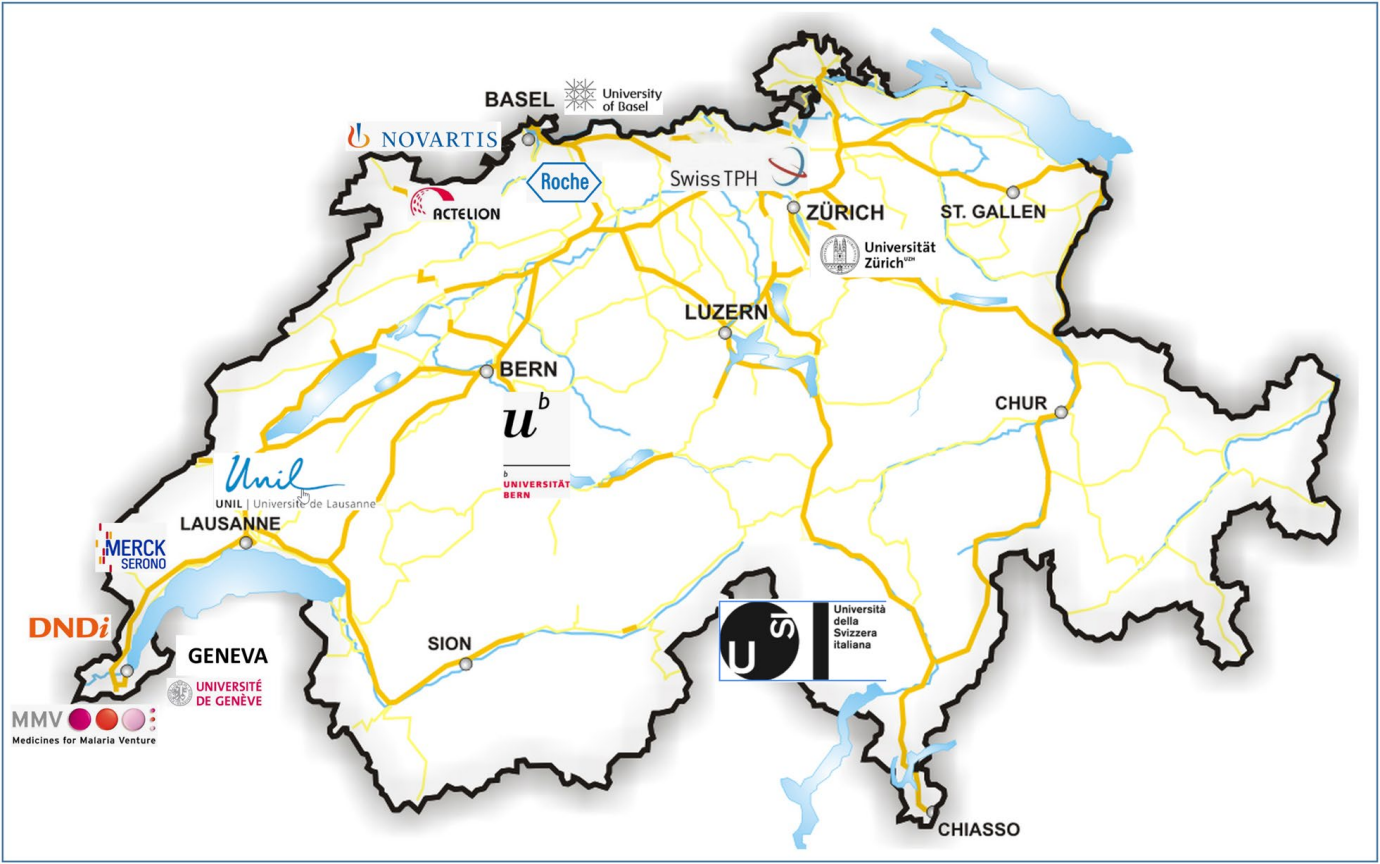
The global portfolio of anti-malarial medicines (development pipeline and approved products). For definitions of the target compound profiles (TCPs) and target product profiles (TPPs) see [6, 8, 9]. 60P, 60° Pharmaceuticals; DND*i*, Drugs for Neglected Diseases *initiative*; GSK: GlaxoSmithBeecham; ITM, Institute of Tropical Medicine; MMV, Medicines for Malaria Venture; U.: University. See the MMV website (http://www.mmv.org) for updated version of this Figure

The present pipeline of candidate drugs for malaria, and approved medicines, is shown in Fig. 4, with pipeline molecules from Swiss-based Pharma companies Novartis and Actelion, and preclinical candidates from the Swiss TPH (Swiss Tropical and Public Health Institute). Along with the approved medicines, this Figure illustrates the past and present engagement of Swiss-based R&D.

Mefloquine (Lariam) is an anti-malarial that was discovered and developed in the 1970s by the Walter Reed Army Institute for Medical Research in the U.S., in a response to the numerous casualties from the disease—at one time, 1% of combat troops per day—during the war in Vietnam. Outside its use for the military, mefloquine was marketed worldwide by Hoffmann-La Roche, based in Basel. These days, mefloquine is mainly used as part of a fixed-dose combination (FDC) with artesunate, provided by CIPLA, an Indian-based company. This FDC was originally developed in collaboration with the Switzerland-based DND*i*.

DND*i* also co-developed, with Sanofi, a new FDC for artesunate-amodiaquine (AS-AQ/Winthrop^®^), specifically formulated and packaged for use in children [11], with the PPP model for late-stage drug development.

Sulfadoxine–Pyrimethamine (Fansidar) was developed in the 1960s, also by Hoffmann-La Roche, after resistance against the individual components severely limited their usefulness. Sulfadoxine–pyrimethamine is not used widely anymore for the treatment of malaria, outside of some states in India, where it remains since 2008 as part of the standard of care as a combination therapy with artesunate [12]. However, it has enjoyed a renaissance as part of a programme called Seasonal Malaria Chemoprevention (SMC). Children in parts of the world where malaria outbreaks occur regularly during a season, such as in Africa’s Sahel region, receive a full course of treatment of sulfadoxine–pyrimethamine plus amodiaquine once each month through the rainy season. The WHO estimates that in 2016, 16 million children under 5 years old were protected, with dramatic effects on morbidity and mortality. The drug used in these studies is now manufactured by a Chinese company, Guilin Pharmaceuticals. Swiss companies driving innovation, and then sharing the market responsibilities with other companies once treatment volumes grow is a common theme.

Artemether–lumefantrine is also known as Coartem^®^ and Coartem-D^®^. Novartis, also based in Basel, has taken a leadership role in the development of this new artemisinin-based FDC for the treatment of uncomplicated malaria (such artemisinin-based combination therapy is usually abbreviated as ACT). Artemisinin was originally discovered in the 1970s as the active ingredient in the herb sweet wormwood (*Artemisia annua*) by Youyou Tu at the Chinese Military Academy of Sciences, who shared a 2015 Nobel Prize for her work [13]. In the same ‘Project 523’ the Chinese developed lumefantrine as a next generation treatment, based on the original quinine/chloroquine/plasmochin scaffold. Lumefantrine was in-licensed by Novartis (then Ciba) in 1992, and developed as an FDC. The combination is an important step forwards in public health, since it avoids the possibility that a patient takes only one of the drugs, which is seen as a potential cause of resistance generation. Later, in collaboration with MMV, Novartis developed a child-friendly, dispersible, taste-masked sweetened version, which has since been launched under the brand name Coartem Dispersible^®^. To date, almost 1000 million doses of artemether–lumefantrine have been supplied by Novartis at an affordable price to countries, of which over 350 million are in the paediatric dispersible form. In doing so, they created a viable market, where generic companies are now supplying the majority of treatments.

## The road to new products: new endoperoxides

Although ACT has been the mainstay of public health policy over the last decade [1, 14], the early days of artemisinin use were complicated by extreme price fluctuations, caused by difficulties in estimating supply needs, and also the 18-month lag time needed to grow new *Artemisia* crops, until the active ingredient could be harvested. The cost-effective synthesis of artemisinin has always proved enormously problematic because of the molecule’s highly unusual endoperoxide structure. In 2002, MMV, the Swiss TPH along with Jonathan Vennerstrom at the University of Nebraska, Hugues Matile at Hoffmann-La Roche in Basel, Heinrich Urwyler at Basilea Pharmaceutica in Basel, and Sue and Bill Charman at Monash University in Melbourne set up a collaboration to develop next-generation endoperoxides. The goal was to find a new molecule that was as active as artesunate (an injectable artemisinin derivative), with a fully synthetic scheme, allowing a low cost of goods, and also the potential to increase the duration of action of the treatment. The first compound to emerge from this highly fruitful collaboration was OZ277 (arterolane; [15, 16]). This was taken into full clinical development, and then partnered with the Indian company Ranbaxy. OZ277 has since been launched as a combination with piperaquine as Synriam™, and is used to treat an estimated 1 million patients per year, primarily in India.

The Swiss-Australian-American collaboration also continued to develop the scaffold further, producing a second-generation clinical candidate, OZ439 (artefenomel; [17, 18]). This molecule has the advantage of a much longer plasma residence time, and higher exposure, with the potential of being part of a single-dose cure. Current anti-malarial therapies require 3 days of treatment, and a single-dose cure is seen as having a very clear advantage of allowing directly observed therapy; [8]. The molecule has passed Phase Ia [19], Phase Ib ([20] in malaria infected volunteers, and Phase IIa studies (monotherapy in patients). It is now in Phase IIb clinical trials.

With the emergence of artemisinin resistance, associated with the newly identified *kelch13* mutations [21–23], there was a fear that these new endoperoxides would not retain activity against these mutant strains. However, careful analysis of the in vitro data suggests that while the compounds were optimized to increase stability in the presence of ferrous iron (Fe^2+^) their action against the artesunate-resistant mutant strains was retained [24].

A third-generation endoperoxide, OZ609 (now called MMV052), has also been identified. This has the potential to be fully active against artemisinin-resistant strains [25]. MMV052 is currently in pre-clinical safety testing, with a view to starting human studies in 2019.

## Phenotypic screening finds new starting points

One of the transformative events of the last decade has been the emergence of high-content phenotypic screens that look for inhibitors of blood-stage infection [26]. Until these became widely available in 2007–8, Swiss TPH was the reference centre for WHO/TDR (the Special Programme for Research and Training in Tropical Diseases) testing, with manual assays allowing the evaluation of around 10,000 compounds per year. The development of ultra-high throughput assays by Case W. MacNamara and Elizabeth Winzeler (at the time part of Novartis’ Genome Research Foundation in San Diego), allowed this number to be increased to several million per year. Novartis was, therefore, the first company to test their entire deck of compounds for potential malaria starting points [27], and set a trend that was followed by GlaxoSmithKline [28], St. Jude Children’s Research Hospital in Memphis Tennessee [29], The Eskitis Institute, Brisbane [30] and others, such as AstraZeneca, Pfizer, Sanofi and several Japanese companies. The early results were impressive, showing a 0.5% hit rate of compounds with an IC_50_ below 2 μM as cut-off, leading to almost 30,000 starting points [26].

From these early starting points, Novartis (through its Institute for Tropical Diseases in Singapore led by Alex Matter, as well as the Genomics campus, and the central facilities in Basel) was able to partner with Swiss TPH, MMV and the UK’s Wellcome Trust for the identification and work-up of two new classes of compounds: KAE609 and KAF156.

KAE609 (cipargamin; formerly NITD609) is a new synthetic anti-malarial spiroindolone analogue that rapidly cleared both major malaria parasites, *Plasmodium falciparum* and *Plasmodium vivax*, in patients when tested in a Phase IIa study [31, 32]. Like all such new molecules it is further tested in the clinic in combinations, to prevent (delay) the emergence of resistance and to reduce the risk of recrudescence in patients. KAF156 also belongs to a completely new class of anti-malarial agents (imidazolopiperazines; [33]). It recently completed Phase I [34, 35] and Phase IIa [36] monotherapy studies.

The results of these massive screening campaigns also benefit drug discovery for other infectious diseases; as part of its open-source drug discovery activities MMV has made key compound sets available for screening on other pathogens, sending out 400 compounds in ‘Malaria Box’ copies to over 200 research teams and overseeing the data reporting [37]. This highly successful initiative was followed by the Pathogen Box [38, 39], and in January 2019, MMV and DND*i* announced the public release of the Pandemic Response Box, drug screening sets against an increasingly wider circle of human pathogens.

ACT-451840 is another anti-malarial candidate with novel structure, discovered in a fully Swiss collaboration between Actelion and the Swiss TPH ([40, 41]; Actelion Pharmaceuticals Ltd is based in Allschwil, Switzerland). The molecule was well tolerated in a Phase Ia study [42], and with further support from MMV demonstrated efficacy in volunteers carrying experimental malaria infection [43].

## Underpinning the technology

Beyond the molecules and clinical candidates where Switzerland has taken the leading role, Swiss organizations have also provided critical resources for most of the early discovery portfolio. One key example in this area is the development of MMV048 (MMV390048; [44], a phosphoinositol (PI) 4-kinase inhibitor discovered by an international team led by Dr Kelly Chibale of the University of Cape Town, South Africa. Here, the Swiss TPH played a key role in the early characterization of the compound series both in vitro and in vivo in the murine models [45]. However, the work to facilitate a technology transfer of the assays to South Africa is equally important, allowing the South African consortium to become fully independent after the first few years and pursue the discovery of additional new molecules [46].

The role of the Swiss-supported clinical centre in Bagamoyo, Tanzania, is another example of this support and empowerment. The Bagamoyo centre is a branch of the Ifakara Health Institute (IHI) that emerged from the Swiss Tropical Institute Field Laboratory created in the middle of the last century by funding from the Basel Foundation and Swiss TPH. Its leadership has always centred on local empowerment, and the centre has been become one of the few Phase I trial sites in sub-Saharan Africa and, overall, one of the most productive clinical trial sites in Africa, directly and indirectly contributing to a decrease in malaria.

## Conclusions

The war against malaria takes place in battles that are hard won (with new drugs and other interventions through integrated approaches) but also lost (emerging resistance, mismanagement of resources, lapses in local healthcare under economic hardship or strife; [47]); outwitting the enemy requires continuous effort. Switzerland’s successes in helping discover and introduce new medicines for this disease are driven by a number of circumstances: the country’s scientific excellence and openness; its highly innovative pharmaceutical industry with a willingness to also invest in ‘diseases of poverty’; its long-standing commitment to promoting universal health as a basic human right; and its hosting of both international organizations that pursue this aim as well as not-for-profit PPPs that have the expertise to align efforts in this area.
